# Retrospective review comparing intrapleural fibrinolytic therapy (alteplase) and surgical intervention in complex pleural effusion

**DOI:** 10.1186/s12890-022-02239-w

**Published:** 2022-11-23

**Authors:** Mohamed Faisal Abdul Hamid, Ahmad Hadyan Husainy Hasbullah, Mas Fazlin Mohamad Jailaini, Nik Nuratiqah Nik Abeed, Boon Hau Ng, Hairulfaizi Haron, Nur Ayub Md Ali, Muhammad Ishamuddin Ismail, Nik Azuan Nik Ismail, Mohd Ramzisham Abdul Rahman, Andrea Yu-Lin Ban

**Affiliations:** 1 Respiratory Unit, Department of Medicine, Faculty of Medicine, Universiti Kebangsaan Malaysia Medical Centre (UKMMC), Jalan Yaacob Latif, Bandar Tun Razak, Cheras, 56000 Kuala Lumpur, Malaysia; 2 Cardiothoracic Unit, Department of Surgery, Faculty of Medicine, Universiti Kebangsaan Malaysia Medical Centre (UKMMC), Jalan Yaacob Latiff, Bandar Tun Razak, Cheras, 56000 Kuala Lumpur, Malaysia; 3 Department of Radiology, Faculty of Medicine, Universiti Kebangsaan Malaysia Medical Centre (UKMMC), Jalan Yaacob Latiff, Bandar Tun Razak, Cheras, 56000 Kuala Lumpur, Malaysia; 4 Heart and Lung Institute, Faculty of Medicine, Universiti Kebangsaan Malaysia Medical Centre (UKMMC), Jalan Yaacob Latiff, Bandar Tun Razak, Cheras, 56000 Kuala Lumpur, Malaysia

**Keywords:** Thoracic surgery, Respiratory infection, Clinical respiratory medicine, Pleural disease

## Abstract

**Background:**

Intrapleural fibrinolytic therapy (IPFT) is one of the treatment options for complex pleural effusion. In this study, the IPFT agent used was alteplase, a tissue plasminogen activator (t-PA). This study aims to determine the difference in the outcome of patients with complex pleural effusion between IPFT and surgery in terms of radiological improvement, inflammatory parameters, length of stay, and post-intervention complications.

**Methods:**

A retrospective review of patients with complex pleural effusion treated at Universiti Kebangsaan Malaysia Medical Center from January 2012 to August 2020 was performed. Patient demographics, chest imaging, drainage chart, inflammatory parameters, length of hospital stay, and post-intervention and outcome were analyzed.

**Results:**

Fifty-eight patients were identified (surgical intervention, *n* = 18; 31% and IPFT, *n* = 40, 69%). The mean age was 51.7 ± 18.2 years. Indication for surgical intervention was pleural infection (*n* = 18; 100%), and MPE (*n* = 0). Indications for IPFT was pleural infection (*n* = 30; 75%) and MPE (*n* = 10; 25%). The dosages of t-PA were one to five doses of 2–50 mg. The baseline chest radiograph in the IPFT group was worse than in the surgical intervention group. (119.96 ± 56.05 vs. 78.19 ± 55.6; *p* = 0.029) At week 1, the radiological success rate for IPFT and surgical intervention were 27% and 20%, respectively, and at weeks 4–8, the success rate was 56% and 80% respectively. IPFT was associated with lesser complications; fever (17.5%), chest pain (10%), and non-life-threatening bleeding (5%).

**Conclusion:**

IPFT was comparable to surgery in radiological outcome, inflammatory parameters, and length of stay with lesser reported complications.

## Background

Pleural effusion is a common medical problem, with varied treatment options depending on the cause. [1] The two commonest causes of pleural effusion are parapneumonic and malignant effusion, with an estimated annual incidence of 300,000 and 200,000 worldwide [2].

Complex pleural effusion is an exudative pleural effusion which are septated, multiloculated or hyperechoic on ultrasound of the thorax [3, 4]. Treatment of infective complex pleural effusion includes antibiotics, pleural fluid drainage, fibrinolytic therapy, and surgery [5]. The option after failed drainage and intravenous antibiotics is surgical intervention [6, 7].

However, a non-invasive strategy such as intrapleural fibrinolytic therapy (IPFT) has been used as an alternative, especially in those not eligible for surgery. IPFT agents include streptokinase, urokinase, alteplase, alteplase with DNase, and tenecteplase. The two most prominent studies on IPFT are Multicenter Intrapleural Sepsis Trial-1 (MIST-1) and Multicenter Intrapleural Sepsis Trial-2 (MIST-2) [8, 9]. The MIST-1 trial did not favor the usage of streptokinase; MIST-2 trial showed that a combination of intrapleural tissue plasminogen activator (t-PA) and dornase alfa therapy improved fluid drainage and reduced frequency of surgical referral and duration of hospital stay. The study also showed DNase or t-PA alone to be ineffective [9].

In 2003, the first successful intrapleural fibrinolytic therapy (IPFT) using intrapleural alteplase was reported [10]. A recent study in 2017 on alteplase alone showed good radiological outcomes [11]. However, pleural hemorrhage has been reported in the literature [12–15]. In centres with difficult access to dornase alfa, the use of alteplase as a single agent has shown promising results [11, 16–18]. Alteplase has been used as IPFT in malignant pleural effusion since 2004 [17].

At Universiti Kebangsaan Malaysia Medical Center (UKMMC), IPFT using alteplase was introduced in 2017 and has been a treatment option for complex pleural effusion (without infection) [19]. In our centre, intrapleural dornase alfa (Pulmozyme) in combination with alteplase was subsequently introduced in 2019 for the management of pleural infection [20]. No data has been compiled for fibrinolysis of complex pleural effusion in the Malaysian population compared to surgery. Hence this inspired us to analyze data on intrapleural alteplase alone in the management of complex effusion before the availability of dornase alfa in our centre.

This study aims to determine the difference in the outcome of patients with complex pleural effusion between IPFT and surgery in terms of radiological improvement, inflammatory parameters, length of stay, and post-intervention complications.

## Methods

This was a retrospective cross-sectional study involving patients admitted to UKMMC with the diagnosis of complex pleural effusion who underwent either IPFT using alteplase (Actilyse, Boehringer Ingelheim) or surgical intervention from January 2012 to August 2020.

The study was approved by the Ethics and Research Committee, Faculty of Medicine, Universiti Kebangsaan Malaysia, with project code no: FF-2020-143.

In this study, complex pleural effusion was defined as the presence of fibrin strands or septations within the pleural cavity, which was proven by thoracic ultrasound. Complex effusions were further categorized into two etiologies; pleural infection and malignant pleural effusion (MPE). Diagnosis of pleural infection was based on clinical suspicion; or pleural fluid that fulfilled at least one of the characteristics: frank pus, exudative nature (according to light's criteria), gram stain or culture positive, lactate dehydrogenase (LDH) > 1000 U/L, pH < 7.2 and/or glucose level < 3.3 mmol/L. MPE is defined as histologically/cytologically proven pleural malignancy or an otherwise unexplained pleural effusion in the context of clinically proven cancer elsewhere.

All patients underwent ultrasound-guided intercostal chest catheter (ICC) placement. A baseline chest radiograph was performed 24 h before IPFT to ensure the ICC position. Alteplase was diluted in 50 mL of 0.9% sodium chloride solution. Intrapleural alteplase was allowed to dwell for 45 min and then unclamped to allow free drainage for 45 min. The same procedure was then repeated 12 h apart. The dose and frequency of instillation depends on physician’s judgement; and clinical and thoracic sonographic improvement. This was assessed on a daily basis. Intravenous tramadol 50 mg was administered before intrapleural alteplase as pre-medication analgesia.

Since 2017, all patients with complex pleural effusion in our centre receive IPFT as first line, except patients who presented with organized stage of empyema as these are referred to surgery. Patients who failed treatment with IPFT are also referred to surgery.

Inclusion criteria are as follows: Patients aged 18 years and above with complex pleural effusion who underwent either IPFT or surgical intervention. Patients diagnosed with complex pleural effusion or empyema were identified from the respiratory unit patient registry, pharmacy list of patients with intrapleural alteplase usage and cardiothoracic unit inpatient census. Patients with incomplete relevant data, e.g. (unavailability of patient’s file, chest radiograph, and biochemical parameters) were excluded from the study.

Serial chest radiographs pre-, and post-intervention (IPFT and surgical intervention) were assessed by a trained radiologist blinded to the outcome. A comparison was made between baseline, at week one post-intervention and chest radiographs at weeks 4–8 post-intervention. The software used was Horos version 3.3.2. The percentage change in chest radiograph was calculated by dividing the calculated reduction of chest radiograph opacity post-intervention (cm^2^) by baseline chest radiograph (cm^2^). The negative percentage indicates worsening opacity post-intervention. Successful intrapleural alteplase instillation/surgical intervention were defined as at least 50% reduction of pleural opacity on chest radiograph after intervention.

The baseline demographic data and common symptoms were collected. Additional efficacy outcomes included the duration of chest tubes in situ, the need for surgical intervention in the IPFT group, length of hospital stay, post-intervention complications, and mortality. Safety was assessed by the frequency and severity of reported adverse events.

All data were analyzed using Statistical Package for Social Sciences (SPSS) version 25. The continuous variables were tested with student t-test for normal distribution and Mann–Whitney u test for non-normal distribution to compare between two groups. The categorical data were tested with Chi square test and Fisher exact test. Significance result was established at *p* < 0.05.

## Results

There were 104 medical records screened between January 2012 and August 2020 of whom eight patients were excluded due to incomplete data, 4 refused surgical intervention (before the availability of IPFT), two resolved with percutaneous drainage, and 32 due to the combination of t-PA and dornase alfa. The final sample, therefore, consisted of 58 patients.

**Fig. 1:**
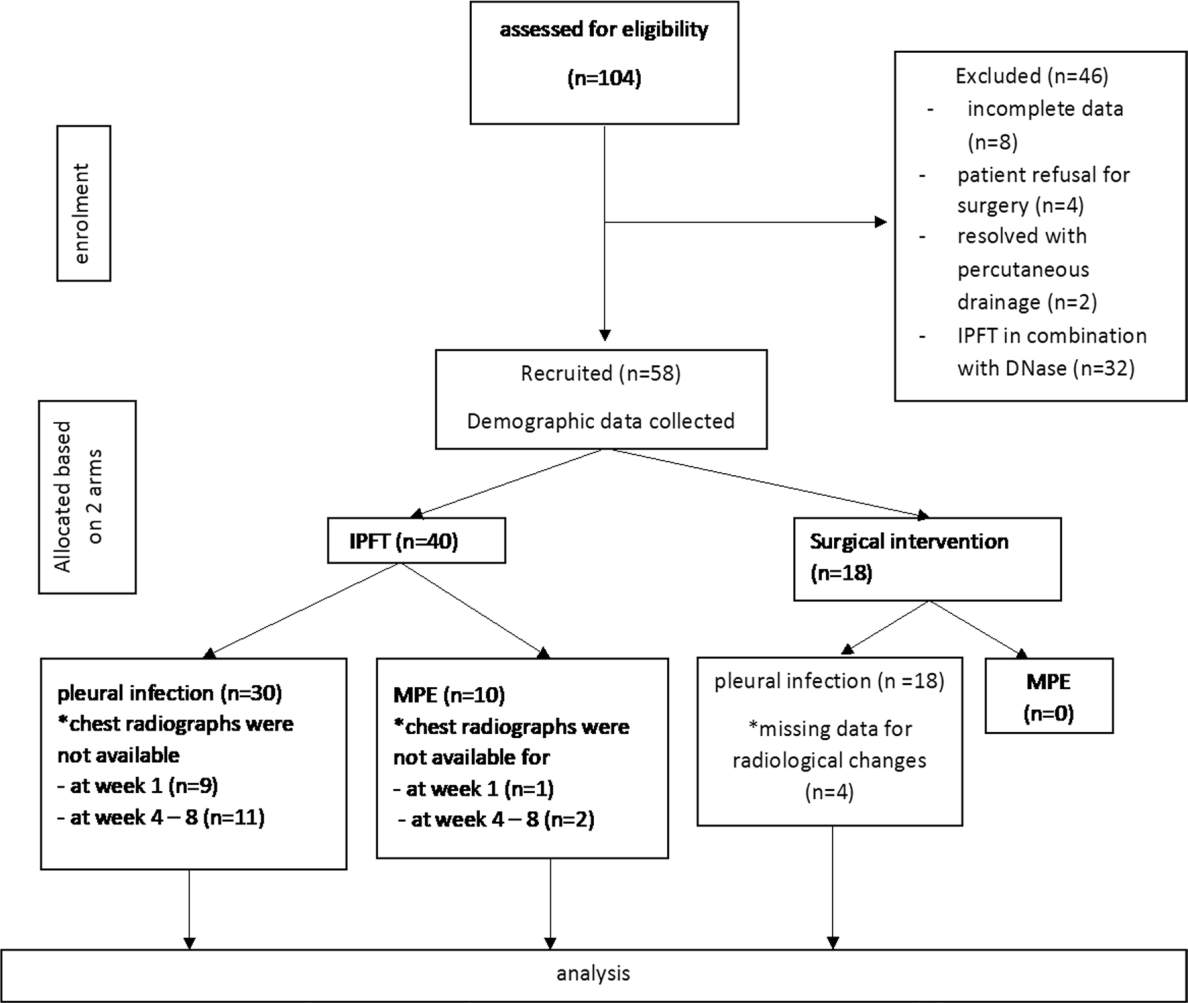
Study flow chart

Forty patients received IPFT (alteplase), and 18 underwent surgical intervention. Of 40 recruited in the IPFT group, 30 were infective and ten malignant. All patients in the surgical intervention group were infective. (Fig. 1).

### Primary outcome

#### IPFT vs. surgery

Patients had a mean age of 51.7 ± 18.2 years, and most were males (64%). There was a significant mean age difference between IPFT and the surgical intervention group. (55.18 ± 18.6 vs. 44 ± 15.1; *p* = 0.029) (Table 1).

**Table 1:** Baseline demographic data comparing IPFT and surgical intervention

	Total population (*n* = 58)	IPFT (*n* = 40)	Surgery (*n* = 18)	*p*- value
Age (years old)—mean	51.7 ± 18.2	55.18 ± 18.6	44 ± 15.1	0.029
*Gender*				
Male	37 (64%)	24 (60%)	13 (72%)	0.37
Female	21 (36%)	16 (40%)	5 (28%)	
*Ethnicity*				
Malay	30 (52%)	18 (45%)	12 (67%)	0.183
Chinese	23 (40%)	17 (42.5%)	6 (33%)	
Indian	5 (8%)	5 (12.5%)	0 (0%)	
*Marital status*				
Single	11 (19%)	6 (15%)	5 (28%)	0.408
Married	40 (69%)	28 (70%)	12 (67%)	
Widow/divorcee	7 (12%)	6 (15%)	1 (6%)	
*Comorbidities*				
DM	18 (31%)	11 (27.5%)	7 (39%)	0.54
Hypertension	26 (45%)	19 (47.5%)	7 (39%)	0.581
Cardiac disease	10 (17%)	7 (17.5%)	3 (17%)	1.000
Lung disease^a^	9 (16%)	5 (12.5%)	4 (22%)	0.438
Liver disease	3 (5%)	1 (2.5%)	2 (11%)	0.225
Kidney disease	8 (14%)	7 (17.5%)	1 (5%)	0.413
Stroke	5 (9%)	5 (12.5%)	0 (0%)	0.311
Autoimmune^b^	2 (3%)	2 (5%)	0 (0%)	1.000
Underlying cancer^c^	8 (14%)	8 (20%)	0 (0%)	0.048
Smoker	22 (38%)	12 (30%)	10 (55%)	0.168
Alcohol	9 (16%)	5 (12.5%)	4 (22%)	0.438

^a^Lung disease: COPD, bronchial asthma

^b^Autoimmune: SLE, Crohn’s disease

^c^Lung cancer, breast cancer, esophageal cancer, renal cell carcinoma

The commonest race was Malays (52%) followed by Chinese (40%) and Indians (8%). Most of them were married (69%). Using Fisher exact test, there was no significant difference in ethnicity and marital status. (Table 1).

The commonest comorbidity was hypertension(45%), followed by diabetes mellitus (31%), cardiac disease (17%), lung disease (16%), kidney disease (14%), underlying cancer (14%), stroke (9%), liver disease (5%) and autoimmune (3%). Thirty-eight per cent were active smokers or ex-smokers, and 16% were alcohol drinkers. (Table 1).

Baseline pleural opacities on chest radiograph in IPFT group were worse compared to surgical intervention (119.96 cm^2^ ± 56.05 vs. 78.19 cm^2^ ± 55.6; *p* = 0.026) with no significant difference in the number of days from the initial chest radiograph to intervention. At week one post-intervention, the chest radiograph changes showed no significant difference between both arms. At weeks 4–8, the chest radiograph changes in the surgical intervention showed greater improvement than the IPFT group. However, it was not statistically significant. IPFT had fewer post-intervention complications compared to the surgical intervention group; *n* = 11 (27.5%) vs. n = 17 (94%); *p* < 0.0005. Other outcome parameters were not significant. (Table 2).

**Table 2:** Outcome of IPFT and surgical intervention

Outcome	IPFT (*n* = 40)	Surgical intervention (*n* = 18)	*p*-value
Baseline chest radiography (cm^2^)—mean	119.96 ± 56.05	78.19 ± 55.6	0.026
Duration from baseline chest radiography to intervention (days)—median (IQR)	2.0 (1–11)	3.5 (1–32)	0.12
*Week 1*^a^			
Chest radiograph changes (%)—mean	31.68 ± 36.37	32.05 ± 33.24	0.976
Success rate (*n*)	8 (27%)	2 (20%)	1.000
*Week 4–8*^b^			
Chest radiograph changes (%)- median (IQR)	49.8 (− 123.48–100)	73.46 (45.79–100)	0.054
Success rate (*n*)	15 (56%)	8 (80%)	0.26
Pre-intervention total white cells (× 10^9^/L)–median (IQR)	10.4 (4.1–60.4)	9.75 (6.9–18.8)	0.545
48 h post-intervention total white cells (× 10^9^/L)—median (IQR)	12.15 (5.4–84.8)	11.1 (4.5–22.8)	0.656
pre-intervention CRP (mg/dL)—median (IQR)	9.64 (0.48–33.2)	9.48 (0.48–43.59)	0.808
48 h post-intervention CRP (mg/dL)—median (IQR)	10.41 (0.77–25.45)	6.41 (2.58–31.84)	0.664
Duration of a chest tube in situ (days)—median (IQR)	14 (3–193)	13.5 (8.5–19.75)	0.686
Length of stay (days)—median (IQR)	21.5 (3–73)	26.5 (7–68)	0.068
Mortality at 30 days (*n*)	2 (5%)	0 (0%)	0.587
Immediate post-intervention complications^c^ (*n*)	11 (27.5%)	17 (94%)	< 0.0005
Hypotension	0	16	
Bleeding	2	1	
Fever	6	0	
Chest pain	3	0	

^a^Missing data: 9 from intrapleural alteplase and 8 from surgical intervention at week 1 (4 pneumonectomies, 1 lobectomy)

^b^Missing data: 11 from intrapleural alteplase and 8 from surgical intervention at weeks 4–8 (4 pneumonectomies, 1 lobectomy)

^c^Immediate post-intervention complications: fever, chest pain, bleeding, hypotension

### Secondary outcome

#### IPFT vs surgery in pleural infection

A separate analysis comparing IPFT and surgical intervention among patients with pleural infection was done. The chest radiograph changes of surgical intervention (32.05% at week 1 and 80% at week 4–8) were insignificant compared to IPFT (32.33% at week 1 and 57.9% at week 4–8). (Table 3).

**Table 3:** Outcome of IPFT and surgical intervention in pleural infection

Outcome	IPFT (*n* = 30)	Surgical intervention (*n* = 18)	*p*-value
Baseline chest radiograph(cm^2^)—mean	112.27 ± 56.31	78.19 ± 55.6	0.087
Duration from baseline chest radiograph to intervention (days)—median (IQR)	2.5 (1–11)	3.5 (1–32)	0.213
*Week 1*^a^			
Chest radiograph changes (%)–mean	32.33 ± 42.84	32.05 ± 33.24	0.985
Success rate (*n*)	8 (38.1%)	2 (20%)	0.428
Week 4–8^b^			
Chest radiograph changes (%)—median (IQR)	62 (− 123.48–100)	73.46 (45.79–100)	0.164
Success rate (*n*)	11 (57.9%)	8 (80%)	0.414
Pre-intervention total white cells (× 10^9^/L)—median (IQR)	9.7 (4.1–31.3)	9.75 (6.9–18.8)	0.881
48 h post-intervention total white cells (× 10^9^/L)—mean	11.59 ± 4.27	12.34 ± 4.97	0.631
Pre-intervention CRP(mg/dL)—median (IQR)	10.03 (0.89–33.2)	9.48 (0.48–43.59)	0.641
48 h post-intervention CRP (mg/dL)—median (IQR)	9.66 (0.77–25.45)	6.41 (2.58–31.84)	0.867
Duration of chest tube in situ (days) median (IQR)	11 (8–23.75)	13.5 (8.5–19.75)	0.983
Length of stay (days)—median (IQR)	20.5 (15–34.75)	26.5 (21.25–40.75)	0.141
Mortality at 30 days(*n*)	1 (3%)	0 (0%)	0.376
Immediate post-intervention complications (*n*)^c^	6 (20%)	17 (94%)	< 0.0005
Hypotension	0	16	
Bleeding	0	1	
Fever	5	0	
Chest pain	1	0	

^a^Missing data: 9 from intrapleural alteplase and 8 from surgical intervention at week 1 (4 pneumonectomies, 1 lobectomy)

^b^Missing data: 11 from intrapleural alteplase and 8 from surgical intervention at weeks 4–8 (4 pneumonectomies, 1 lobectomy)

^c^Immediate post-intervention complications: fever, chest pain, bleeding, hypotension

The number of cases with post-intervention complications in the IPFT group was 6 (20%) compared to 17 (94%) surgical patients with post-operative complications*.* (Table 3).

#### Pleural infection vs. MPE in IPFT group

The baseline chest radiograph was similar in pleural infection and MPE, with no significant difference. The MPE group showed a 0% success rate at week 1. Both pleural infection and MPE did not require surgical intervention post-IPFT. MPE showed higher total white cells as compared to pleural infection pre- and post-intervention (9.7 × 10^9^/L (4.1–31.3) vs. 12.8 × 10^9^/L (9.1–60.4); *p* = 0.023 and 11.2 × 10^9^/L (5.4–25) vs. 15.6 × 10^9^/L (12–84.8); *p* = 0.001). (Table 4). There was no recurrence of MPE between week 1 and weeks 4–8 post-intervention.

**Table 4:** Outcomes of intrapleural alteplase in infective and malignant pleural effusion

Outcome	pleural infection (*n* = 30)	MPE (*n* = 10)	*p-*value
Baseline chest radiograph (cm^2^)	112.26 ± 56.31	137.91 ± 54.2	0.258
Duration from baseline chest radiograph to intervention (days)	2.5 (1–11)	1 (1–8)	0.319
*Week 1*^a^			
Chest radiograph changes (%)–mean	32.33 ± 42.85	30.14 ± 14.25	0.883
Success rate (n)	8 (38.1%)	0 (0%)	-
*Week 4–8*^b^			
Chest radiograph changes (%)—median (IQR)	62 (− 0.5–96.8)	37.02 (0.83–68.42)	0.457
Success rate(*n*)	11 (57.9%)	4 (50%)	1.000
Pre-intervention total white cells (× 10^9^/L)—median (IQR)	9.7 (4.1–31.3)	12.8 (9.1–60.4)	0.023
48 h post-intervention total white cells (× 10^9^/L)—median (IQR)	11.2 (5.4–25)	15.6 (12–84.8)	0.001
Pre-intervention CRP (mg/dL)—median (IQR)	10.03 (0.89–33.2)	8.2 (0.48–17.10)	0.210
48 h post-intervention CRP (mg/dL)–median (IQR)	11.59 ± 4.27	27.9 ± 24.24	0.079
Increment in drainage post-intervention (mls)—median (IQR)	1190 (− 21.75–2414.5)	1020 (− 72.5–2392.5)	0.851
Duration of chest tube (days)—median (IQR)	11 (8–23.75)	20 (11.5–23)	0.404
Length of stay (days)—median (IQR)	20.5 (15–34.75)	21.5 (16.5–26)	0.719
Surgical referral (*n*)	0 (0%)	0 (0%)	-
Mortality in 30 days (*n*)	1 (3%)	1 (10%)	0.336
Immediate post-intervention complications^c^ (*n*)	6 (20%)	5 (50%)	0.103
Bleeding	0	1	
Fever	5	1	
Chest pain	1	3	

^a^ missing data: 9 for infective pleural effusion and 1 for malignant pleural effusion

^b^ missing data: 11 for infective pleural effusion and 2 for malignant pleural effusion

^c^ immediate post intervention complications: bleeding, fever and chest pain

The commonest IPFT regime was 10 mg × 5 doses, given 12 h apart (52.5%), with different dosages in other regimes ranging from 2 mg × 2 doses to a single dose of 50 mg. (Table 5).

**Table 5:** Details of alteplase regime given

Intrapleural alteplase dosages (mg)	Frequency (*n* = 40)
50 × 1	1 (2.5%)
10 × 5	21 (52.5%)
10 × 4	2 (5%)
10 × 3	2 (5%)
5 × 5	2 (5%)
5 × 4	2 (5%)
5 × 1	2 (5%)
2.5 × 1	2 (5%)
16 × 3	1 (2.5%)
10 × 1	1 (2.5%)
5 mg × 1,10 mg × 2	1 (2.5%)
5 × 3	1 (2.5%)
2.5 × 3	1 (2.5%)
2 × 1	1 (2.5%)

The groups were divided into two; (50 mg or less than 50 mg). At week 1, the overall success rate for pleural infection was 38.1%, and 57.9% after 4–8 weeks, and for MPE was 0% at week 1, and 50% at weeks 4–8. However, there was no significant difference between the two different dosages. (*p* = 1.000) (Table 6).

**Table 6:** The success rate of alteplase regime

Accumulative dosages	Pleural infection (*n* = 21)		MPE (*n* = 9)	
	Week 1^a^	Week 4–8^b^	Week 1	Week 4–8^c^
Less than 50 mg	3/8 (37.5%)	3/6 (50%)	0 (0%)	3/5 (60%)
50 mg	5/13 (38.46%)	8/13 (61.5%)	0 (0%)	1/3 (33.3%)
Total (N)	8 /21 (38.1%)	11/19 (57.9%)	0%	4/8 (50%)
*p* value	1.000	1.000	–	1.000

^a^9 missing data at week 1

^b^11 missing data at weeks 4–8

^c^2 missing data at weeks 4–8

### Adverse effects of IPFT and surgical intervention

The commonest adverse effects of IPFT in pleural infection were fever, followed by chest pain and bleeding, while in MPE, the commonest adverse effects were chest pain, followed by fever and bleeding.

Most surgical patients underwent decortication (72%), with only 22% converted to pneumonectomy, and another 6% underwent lobectomy. Sixteen (89%) patients developed peri- or post-operative hypotension requiring inotropic support while another 12 (67%) had peri- or post-operative bleeding requiring blood transfusion.

There were two deaths in IPFT group (1 pleural infection, 1 MPE). On further analysis, one patient in the pleural infection group died at day 25 post-IPFT due to nosocomial infection with the multidrug-resistant organism (MRO) *Acinetobacter* sp septicemia. In comparison, 1 patient in the MPE group died of progression of advanced lung carcinoma at day 21 post-intervention. There were no reported deaths in the surgical intervention group.

A few cases in the surgical arm needed to be converted to pneumonectomy (*n* = 4) and lobectomy *(n* = 1). This was due to the extension of the empyema into the lung parenchyma resulting in a considerable lung abscess, which resulted in decortication with lobectomy/pneumonectomy.

## Discussion

To the best of our knowledge, this is the first local study looking at the outcomes of intrapleural alteplase as the choice of IPFT for complex pleural effusion compared to surgical intervention. There are other studies/case reports in our country on different regiments of alteplase with/out dornase alfa [20–24].

There were more patients in the IPFT group compared to the surgical intervention group (n = 40 vs. n = 18). The smaller number in the surgical intervention group could be due to the total usage of IPFT. In 2017, IPFT was first introduced in our centre, as the first-line treatment for complex pleural effusion. Since 2017, none of the patients who underwent IPFT required surgical intervention.

Surgical intervention has been reported to have higher success rates compared to IPFT [25]. The IPFT used in that study was streptokinase. Local data for outcomes of alteplase compared to surgery is limited.

Our study found that the post-intervention chest radiograph changes in IPFT were lower than in the surgical intervention group. The baseline chest radiographs in IPFT were worse than in the surgical intervention group. We excluded patients who had undergone pneumonectomy and lobectomy from our radiographic analysis as post intervention (surgical) chest radiographs have been reported to show more pleural effusion [26]. Twenty-two per cent of surgical patients were converted to pneumonectomy on the table.

The success rate for IPFT was 57.9% which is lower than various studies, between 80 and 90%. [11, 16–18, 27–29] This is due to the patient profile difference and the smaller sample size. In addition, we encountered missing data, for the post IPFT radiological improvement. Between the years 2017 to 2018, there was a problem with the radiology PAX server in UKMMC, resulting in some files being corrupted. Some patients did not repeat chest radiographs at weeks 4 to 8. These patients defaulted on their follow-up or chose to be followed up at another hospital.

There was no statistical difference in the median length of stay between IPFT (21.5 (3–73) days) and the surgical intervention group (26.5 (7–68) days). Our study’s median duration of intravenous antibiotics was 33.5 (33–75) days. The median duration of intravenous antibiotics may not reflect treatment duration for pleural infection as data collected included total antibiotics used for other indications. Most patients developed nosocomial infections (hospital-acquired pneumonia, thrombophlebitis, urinary tract infection) requiring antibiotics therapy.

Generally, our patients had relatively low white cell count and CRP. This can be explained by two possible reasons: (1) The values were measured prior to intervention and not on day 1 of admission. Hence, some of the patients had already received intravenous antibiotics which may have reduced the white cell count. (2) Patients with tuberculosis (TB) were also included, and patients with pleural TB have relatively low white cell count to begin with."

The significant difference in post-intervention complications (hypotension and bleeding) was because the cases in the surgical intervention arm were more complicated than the cases in the IPFT group. 4 cases out of 18 were converted from decortication to pneumonectomy and lobectomy. The 4 cases were 24, 40, 42, and 75 years old with multiple comorbidities.

MPE was diagnosed either by findings of metastases at the pleura on CT scan or pleuroscopy, pleural fluid cytology, or pleural biopsy histopathology. [30–32] There were 4 newly diagnosed lung carcinoma with three confirmed by lung biopsy (2 adenocarcinoma, 1 neuroendocrine carcinoma) and another patient was diagnosed by CT thorax imaging as he declined lung biopsy. Other underlying cancers that came with malignant pleural effusion were two breast cancers, two lung adenocarcinomas, and one each for oesophageal and cervical carcinoma. This finding was similar to previous studies that found the most common primary for malignant pleural effusion was from the lungs, followed by the breast. [33, 34].

Patients with malignant pleural effusion were not referred to surgery due to the advanced stage of cancer, while in pleural infection, there was significant improvement of pleural effusion after fibrinolysis. Longer duration of a chest tube in situ was seen in MPE (median 20 days (11.5–23)) as some of the patients had an indwelling pleural catheter (IPC). The mean duration of the chest drain was long as it was measured from day 1 insertion and not the mean duration of chest drain after intervention. These patients were managed by general medical team and it took few days before referral to the respiratory team was made.

The length of stay was similar between infective (median 20.5 days (15–34.75)) and MPE (median 21.5 days (16.5–26)) partly because of the antibiotics initiated to cover for infection, as evidenced by a high total white cell count. Patients usually have intravenous antibiotics for at least 2–3 weeks. In addition, some patients developed nosocomial infection. The administration of intravenous home antibiotics via peripherally inserted central catheter (PICC) line is not available locally.

While no cases recorded more than 50% reduction in pleural effusion on chest radiograph at week 1, there was a mean reduction of 30.14% ± 14.25, which was not statistically different compared to pleural infection hence the role of the IPFT as a means of reducing the accumulation of pleural fluid in complex pleural effusion. The success rate of IPFT in MPE improved to 50% in weeks 4 to 8, likely due to spontaneous resolution of pleural effusion or initiation of pleurodesis that stopped the re-accumulation of pleural fluid [34, 35]. The prolonged used of antibiotics in the pleural infection group may have contributed to further improvement of pleural opacity.

Our study's most common alteplase regime was 10 mg × 5 doses (52.5%). The decision to use different dosages was dependent on the amount of pleural effusion on chest radiograph and underlying diseases such as liver disease, kidney disease, and anaemia; as well as the concurrent use of anticoagulant.

The improvement of success rate in our study at weeks 4 to 8 (27% to 56%) was due to delayed radiological changes post-intervention. The delayed radiological changes were similar in both surgical intervention and MPE groups.

TB is prevalent in Malaysia, and loculated pleural effusions are a known complication. The etiology is likely due to a delayed hypersensitivity response. Fibrosis of the pleural cavity leads to pleural thickening, and this can compromise pulmonary function [36]. There were 12 patients with tuberculous complex pleural effusion who received IPFT. Of the 12, 1 patient had residual pleural thickening (8%). There is no available data on the role of alteplase in reducing residual pleural thickening, though Cao et al. showed good results with intrapleural urokinase. [37].

Two cases (3%) in the MPE group developed bleeding (intrapleural haemorrhage, haematuria) after IPFT requiring packed cells transfusion. Both patients developed bleeding after completing 5 doses of 10 mg and 5 mg intrapleural alteplase, respectively. None had a hemodynamic compromise. According to the literature, the incidence of bleeding post-IPFT ranges from 1 to 8%. [11, 17, 18, 27–29] Other complications found in our study were fever (17.5%) and chest pain (10%), with the latter being more common in malignant pleural effusion, probably due to non-expandable lung, however, it did not result in early termination of IPFT.

### Limitations

We did not achieve the required number of cases in the surgical intervention arm. Fifteen patients were excluded from radiographic analysis due to corrupted files due to a problem with the PAX server. Another limitation was the inability to differentiate pleural effusion from pleural thickening from chest radiographs alone on follow-up. Furthermore, it would be ideal to have multiple radiologists analyze the images to mitigate bias. We also recognized that in our study, surgical patients were younger. The higher likelihood of comorbidities that comes with increased age may determine outcomes and treatment responses.

## Conclusions

IPFT was comparable to surgical intervention in terms of radiological outcome and length of stay, with fewer post-intervention complications. IPFT in both pleural infection and MPE showed similar radiological improvement, pleural fluid drainage, length of stay, and post-intervention complications.

The use of IPFT is relatively safe, with no significant adverse effects. A multicentre randomized study is needed to further evaluate and determine the safety, efficacy, and optimal doses of intrapleural alteplase compared to surgical intervention.

Overall, this retrospective study describes local outcomes with alteplase and surgery for complex effusions. Its relevance is limited in areas where t-PA and dornase alfa are both available, as data from large dedicated trials have shown the superiority of the combined drugs. The description of local outcomes with t-PA alone may be useful for other areas with similar patient populations where dornase alfa is unavailable.

## Data Availability

The datasets used and/or analyzed during the current study available from the corresponding author on reasonable request.

## Abbreviations

CRP: C-reactive protein
DNase: Deoxyribonuclease
IQR: Interquartile range
IPFT: Intrapleural fibrinolysis therapy
LDH: Lactate dehydrogenase
MPE: Malignant pleural effusion
SD: Standard deviation
TB: Tuberculosis
t-PA: Tissue plasminogen activator
